# Fruit softening: evidence for pectate lyase action *in vivo* in date (*Phoenix dactylifera*) and rosaceous fruit cell walls

**DOI:** 10.1093/aob/mcab072

**Published:** 2021-06-10

**Authors:** Thurayya Z S Al Hinai, Robert A M Vreeburg, C Logan Mackay, Lorna Murray, Ian H Sadler, Stephen C Fry

**Affiliations:** 1 The Edinburgh Cell Wall Group, Institute of Molecular Plant Sciences, The University of Edinburgh, Daniel Rutherford Building, The King’s Buildings, Max Born Crescent, Edinburgh, UK; 2 EastCHEM School of Chemistry, The University of Edinburgh, The King’s Buildings, Edinburgh, UK

**Keywords:** Cell wall, Driselase, fruit softening, high-voltage paper electrophoresis, homogalacturonan, pectate lyase, date (*Phoenix dactylifera*), pear (*Pyrus communis*), rowan (*Sorbus aucuparia*), apple (*Malus pumila*)

## Abstract

**Background and Aims:**

The programmed softening occurring during fruit development requires scission of cell wall polysaccharides, especially pectin. Proposed mechanisms include the action of wall enzymes or hydroxyl radicals. Enzyme activities found in fruit extracts include pectate lyase (PL) and endo-polygalacturonase (EPG), which, *in vitro*, cleave de-esterified homogalacturonan in mid-chain by β-elimination and hydrolysis, respectively. However, the important biological question of whether PL exhibits action *in vivo* had not been tested.

**Methods:**

We developed a method for specifically and sensitively detecting *in-vivo* PL products, based on Driselase digestion of cell wall polysaccharides and detection of the characteristic unsaturated product of PL action.

**Key Results:**

In model *in-vitro* experiments, pectic homogalacturonan that had been partially cleaved by commercial PL was digested to completion with Driselase, releasing an unsaturated disaccharide (‘ΔUA–GalA’), taken as diagnostic of PL action. ΔUA–GalA was separated from saturated oligogalacturonides (EPG products) by electrophoresis, then subjected to thin-layer chromatography (TLC), resolving ΔUA–GalA from higher homologues. The ΔUA–GalA was confirmed as 4-deoxy-β-l-*threo*-hex-4-enopyranuronosyl-(1→4)-d-galacturonic acid by NMR spectroscopy. Driselase digestion of cell walls from ripe fruits of date (*Phoenix dactylifera*), pear (*Pyrus communis*), rowan (*Sorbus aucuparia*) and apple (*Malus pumila*) yielded ΔUA–GalA, demonstrating that PL had been acting *in vivo* in these fruits prior to harvest. Date-derived ΔUA–GalA was verified by negative-mode mass spectrometry, including collision-induced dissociation (CID) fragmentation. The ΔUA–GalA:GalA ratio from ripe dates was roughly 1:20 (mol mol^–1^), indicating that approx. 5 % of the bonds in endogenous homogalacturonan had been cleaved by *in-vivo* PL action.

**Conclusions:**

The results provide the first demonstration that PL, previously known from studies of fruit gene expression, proteomic studies and *in-vitro* enzyme activity, exhibits enzyme action in the walls of soft fruits and may thus be proposed to contribute to fruit softening.

## INTRODUCTION

### Fruit softening in general

The programmed softening that occurs during the ripening of many fruit species requires cell wall loosening and a reduction in cell–cell adhesion as a result of dissolution of the pectin-rich middle lamella (Jarvis *et al.*, 2003; Brummell, 2006). Characteristic modifications include solubilization and depolymerization of pectin, loss of neutral sugars from pectic side chains, cell wall swelling and disassembly of the xyloglucan–cellulose network (Paniagua *et al.*, 2017). These modifications are partly due to non-enzymic reactions with reactive oxygen species (especially the hydroxyl radical, ·OH; Dumville and Fry, 2000; Airianah *et al.*, 2016) or expansins (Brummell *et al.*, 1999), and partly the result of wall-modifying enzymes secreted into the apoplast during ripening. These enzymes act by cleaving polysaccharides, resulting in mechanical weakening. There are three such types of enzyme activity: hydrolases, transglycosylases and lyases, requiring specific substrates (Moya-León *et al.*, 2019). Endo-acting wall-modifying enzymes studied in relation to fruit softening include xyloglucan endotransglucosylase/hydrolases (XTHs) (Saladié *et al.*, 2006; Miedes and Lorences, 2009), cellulases (Dong *et al.*, 2018), endo-polygalacturonases (EPGs) (Wu *et al.*, 1993; Asif and Nath, 2005; Quesada *et al.*, 2009), pectate lyases (PLs) (Marín-Rodríguez *et al.*, 2003; Dong *et al.*, 2018) and rhamnogalacturonan lyases (Ochoa-Jiménez *et al.*, 2018; Méndez-Yañez *et al.*, 2020). In addition, pectin methylesterases (Tieman *et al.*, 1992; Phan *et al.*, 2007) and exo-polygalacturonases (Bartley, 1978; Yang *et al.*, 2018) attack pectin but not by mid-chain cleavage. However, the link between enzyme activities (measured *in vitro* after extraction of the enzymes) and fruit softening was often contradictory.

### Pectins

In tomato, the most extensively studied model fruit, and in many other fleshy fruits, pectin modification is the most pronounced cell wall change during ripening. Pectin has three major domains (reviewed by Fry, 2010): homogalacturonan (HG; ‘pectate’), which consists of a mainly unbranched chain of anionic (1→4)-α-d-galacturonic acid (GalA) residues plus neutral blocks of methyl-esterified (1→4)-α-GalA residues; rhamnogalacturonan-I, which has a backbone of repeating disaccharide units of (1→4)-α-d-GalA-(1→2)-α-l-Rha (where Rha = rhamnose), with neutral side chains of β-galactose and α-arabinose usually attached to approx. 50 % of the rhamnose residues at their *O*-4 position; and rhamnogalacturonan-II, which consists of eight or more (1→4)-α-d-GalA residues as a backbone to which five different side chains are attached, making a highly complicated structure. Another, often minor, domain of pectin is xylogalacturonan which has an α-d-GalA backbone (with or without methyl esters) with β-d-xylose and α-l-fucose side chains. The present study focuses on HG, which is usually the most abundant pectic domain.

### HG-acting enzymes

Plants possess two enzyme activities capable of cleaving the backbone of anionic HG domains in mid-chain: EPG and PL. Both of these act only on anionic HG domains, and therefore prior de-methylesterification by pectin methylesterase may be necessary (Tieman *et al.*, 1992; Dong *et al.*, 2018). In addition, plants have exo-PG (α-d-galacturonidase), which removes GalA residues one at a time from the non-reducing end of HG, presumably having relatively little effect on the cell wall’s mechanical properties. (In this paper, we use ‘EPG’ specifically for endo-polygalacturonase and ‘PG’ for polygalacturonase where we feel the data do not distinguish endo- from exo-.)

**Fig. 1.**
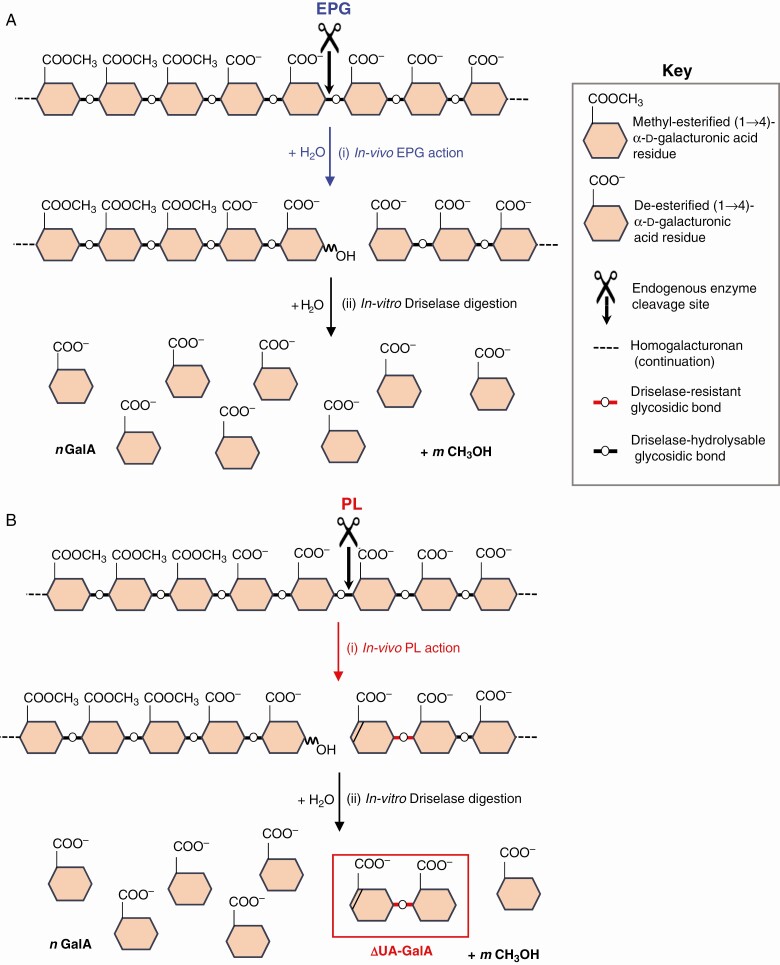
EPG and PL action on homogalacturonan followed by Driselase digestion. (A) EPG attacking the (1→4) glycosidic bond between de-esterified GalA residues of HG, producing a new reducing terminus and a new saturated non-reducing terminus by hydrolysis. Digestion of EPG products with Driselase cleaves the remaining methyl ester groups and the whole chain of HG to monomeric GalA by its combination of hydrolysing enzymes including PME, EPG and galacturonidase. (B) PL attacking the same substrate, producing a new reducing terminus and a new unsaturated non-reducing (ΔUA) terminus by β-elimination. Digestion of PL products with Driselase cleaves the remaining methyl ester groups and the whole chain of HG to GalA monomers plus the dimer, ΔUA–GalA, the unique PL action fingerprint.

EPG, which catalyses endo-hydrolysis (Fig. 1A, reaction i), is the most studied pectin-cleaving enzyme, yet its effect on fruit softening may be low (Wang *et al.*, 2018). Genes encoding EPGs are often upregulated during fruit ripening (Tucker and Grierson, 1982), suggesting that this enzyme may be produced during softening. This is supported by reports of PG activity extractable from fruit (Wu *et al.*, 1993; Orr and Brady, 1993; Villarreal *et al.*, 2008; Zhang *et al.*, 2020). However, many such reports have not satisfactorily distinguished between EPG and PL, and even exo-PG, activities. For example, ‘EPG’ activity in strawberry extracts was often assayed as *in-vitro* production of new reducing termini (i.e. as total reducing groups) from a substrate of pure HG (Villarreal *et al.*, 2009; Figueroa *et al.*, 2010; Zhou *et al.*, 2015, based on an influential study by Gross, 1982); however, reducing groups are generated from HG by endo-PG, exo-PG and PL, and also by ·OH reactions, so these three enzyme activities and the reactive oxygen species would not have been distinguished in such studies.

Transformation experiments with antisense PG genes in tomato and strawberry produced discrepant data. In tomato, PG expression (measured as mRNA levels by northern blotting) could be reduced to 1 % of that of the wild type without affecting softening (Smith *et al.*, 1990; Brummell and Harpster, 2001), whereas in strawberry and apple, firmer fruits were produced when PG expression was reduced to 5–25 % of that of the wild type (Quesada *et al.*, 2009; Atkinson *et al.*, 2012; Posé *et al.*, 2015).

PL cleaves anionic HG domains by a β-elimination reaction (i.e. non-hydrolytically) to give a product with a 4-deoxy-β-l-*threo*-hex-4-enopyranuronosyl residue (abbreviated as ΔUA, for ‘unsaturated uronic acid’) at the newly formed non-reducing end (Fig. 1B, reaction i) (Fuchs, 1965; Shaligram and Singhal, 2010; Nasuno and Starr, 1967; Iqbal *et al.*, 2016; Zhou *et al.*, 2016). (Note: rules of carbohydrate nomenclature dictate that a β-l-ΔUA residue is the product expected when a lyase catalyses an elimination reaction starting with a pectic α-d-GalA residue; this does not imply any change in the configuration at carbon-1.) Earlier work had reported a microbial pectin lyase (not PL) that acts on methylesterified HG (Albersheim *et al.*, 1960). PL gene expression (monitored as mRNA accumulation) has been reported in ripening fruits including strawberry (Benítez-Burraco *et al.*, 2003; Figueroa *et al.*, 2008), banana (Dominguez-Puigjaner *et al.*, 1997; Pua *et al.*, 2001), mango (Chourasia *et al.*, 2006; Deshpande *et al.*, 2017) and grapes (Nunan *et al*., 2020). Despite early negative reports (e.g. Besford and Hobson, 1972), recent studies have suggested a central role for PL genes in tomato fruit softening: tomato fruits with silenced PL genes had reduced PL mRNA expression, reduced extractable PL enzyme activity and increased fruit firmness (Uluisik *et al.*, 2016; Yang *et al.*, 2017). A putative PL gene was ascribed a possible role in softening (Jiménez-Bermúdez *et al.*, 2002; Marín-Rodríguez *et al.*, 2002). PL activity (assayed *in vitro*) is extractable from ripening strawberry (Zhou *et al.*, 2016), banana (Marín-Rodríguez *et al.*, 2003) and several other fruits (Wang *et al.*, 2018).

### Expression, activity and action

Fruit species clearly differ in the reactions modifying HG during ripening, and in no species can the reaction(s) which contribute the ‘key’ role in softening be precisely defined. Often, mRNA accumulation has been taken as evidence of ‘contribution’. Fewer studies have assayed extractable enzyme activities, and very few have tested whether the enzymes exhibit action in the fruit *in vivo*. Activity is measured in katals under optimized conditions *in vitro*; action is what can be observed *in vivo*, in living fruit tissue. Direct evidence for enzyme action can potentially be provided by analysis of changes in polysaccharide chemistry during ripening.

There are several plausible reasons why an enzyme that exhibits *in-vitro* activity when extracted from the plant might not exhibit action within the living plant. For example (Fry, 2004), the enzyme and its substrate may be spatially separated, specific inhibitors may be present, the apoplastic redox potential, pH or ionic strength may not be optimal, or the prior action of a necessary helper enzyme (pectin methylesterase in the case of EPG and PL) may not have occurred.

### Dates

The present work focuses on fruit of the date (*Phoenix dactylifera*), a dioecious monocot in the commelinid family Arecaceae. It is widely cultivated in the Middle East and North Africa. Date ripening in many varieties is marked by a decrease in water content and an increase in soluble sugar (Ahmed *et al.*, 1995; El Arem *et al.*, 2011). A remarkable decrease in the cell wall content of the fruit pulp has also been reported in ripe date as well as other fleshy fruits (Vicente *et al.*, 2007; Griba *et al.*, 2013).

In date fruits, pectin is the major non-cellulosic cell wall component, rather than hemicelluloses as in commelinid grasses. During date ripening, a decreased degree of HG methylesterification was reported (Griba *et al.*, 2013), making it a potential substrate for hydrolysis by PG and β-elimination by PL. Moreover, an increase in extractable cellulase, β-galactosidase (Rastegar *et al.*, 2012) and PG (Serrano *et al.*, 2001) activities was reported in date. β-Galactosidase and PG activities peaked at the full ripe stage, after which the PG activity was reduced while β-galactosidase activity remained high. The increase in the extractable activities of these two enzymes was correlated with fruit softness during ripening (Serrano *et al.*, 2001). No data are available about PL in dates – either PL activity in extractable proteins or PL action *in muro*. We aimed to supply the first evidence for PL action in fruits.

### Strategy for detecting PL action

Each of the proposed mechanisms of HG endo-cleavage leaves a fingerprint on the fruit’s pectin which may be used as a tool to examine the *in-vivo* contribution of each mechanism to ripening. Oxidation by ·OH leaves mid-chain oxo groups (Airianah *et al.*, 2016), hydrolysis by EPG leaves a new non-reducing terminal GalA residue, and β-elimination by PL leaves a new non-reducing terminal ΔUA residue. It had not been tested whether PL exhibits action *in vivo* – in the fruit of any species, or indeed in any other plant organs. Here, we provide the first evidence of PL’s *in-vivo* action by detecting its unique fingerprint (containing ΔUA) in ripe fruits of several species.

## MATERIALS AND METHODS

### Materials

Ripe date (*Phoenix dactylifera* ‘Khalas’) fruits were collected from three randomly selected trees from a date palm field in Oman in June 2018. The samples were stored at −80 °C. Pear (*Pyrus communis* ‘Conference’), rowan (*Sorbus aucuparia*) and apple (*Malus pumila* ‘Bramley’) fruits were collected from a private garden in Edinburgh, UK.

*Cellvibrio japonicus* PL, purchased as an ammonium sulfate suspension (from Megazyme; https://www.megazyme.com), was centrifuged at 14 500 *g* for 3 min and the pellet was re-dissolved in water at 10 U mL^–1^. *Aspergillus aculeatus* EPG, purchased as an ammonium sulfate suspension (Megazyme; https://www.megazyme.com), was not pelletable and was therefore dialysed against pyridine/acetic acid/0.5 % chlorobutanol buffer (1:1:98), then diluted to 10 U mL^–1^. Driselase (from *Basidiomycetes* sp. 067K1303, Sigma) was purified by ammonium sulfate precipitation and gel permeation chromatography (Fry, 2000). Homogalacturonan (= ‘polygalacturonic acid’ or ‘sodium polypectate’), CAPS [3-(cyclohexylamino)-1-​propanesulfonic acid] and CaCl_2_ were from Sigma–Aldrich (https://www.sigmaaldrich.com/united-kingdom.html). Aluminium-backed F254 silica-gel thin-layer chromatography (TLC) plates (1.05554.0001) were from Merck (https://www.merckgroup.com/uk-en). We found that for oligogalacturonide analysis, aluminium-backed plates gave much better chromatography than the corresponding plastic-backed plates.

### *PL* in-vitro *activity products*

A reaction mixture of 6.6 mg mL^–1^ HG, 50 mm CAPS (Na^+^, pH 10), 1 mm CaCl_2_ and 3.3 U mL^–1^ PL was incubated at 20 °C. The reaction was stopped at the desired time points by addition of 0.2 volumes of formic acid. Products were used as (unsaturated) ΔUA–GalA_*n*_ markers.

### *EPG* in-vitro *activity products*

Commercial EPG at 10 U mL^–1^ was used to digest 20 mg mL^–1^ HG in pyridine/acetic acid/0.5 % chlorobutanol buffer (1:1:98), pH 4.7. The reaction mixture was incubated on a wheel at 20 °C overnight and products were used as (saturated) GalA_*n*_ markers.

### Paper chromatography

PL and EPG *in-vitro* digestion products were loaded on Whatman No. 1 paper and run in ethyl acetate/acetic acid/water (EAW) 10:5:6 for 30 h. The paper was then dried and stained with AgNO_3_ (Fry, 2000).

### Preparation of alcohol-insoluble residue (AIR)

We prepared AIR as the source of cell walls by homogenizing (using a pestle and mortar) 9 g of fresh fruit in 36 mL of 75 % ethanol containing 5 % formic acid. The homogenate was incubated on a wheel at 20 °C overnight and then centrifuged at 3220 *g* for 5 min. The pellet was washed twice in 75 % ethanol and then saponified in 10 mL of aqueous 0.2 m Na_2_CO_3_ at 4 °C for 16 h. The Na_2_CO_3_ was neutralized by acetic acid, then ethanol was added to a final concentration of 75 % and the suspension was kept overnight at 4 °C (thus any polysaccharides solubilized in Na_2_CO_3_ would be reunited with the insoluble wall fraction). The mixture was centrifuged at 3220 *g* for 5 min. The pellet was washed three times in 75 % ethanol and twice in acetone for 1 h each on a wheel. The final pellet of (de-esterified) AIR was dried and stored at room temperature for analysis.

### Driselase digestion

De-esterified date AIR (25 mg d. wt) was digested in 3 mL of 0.05 % Driselase in pyridine/acetic acid/water (1:1:98 v/v/v, containing 0.5 % chlorobutanol) at 37 °C for 3 d. Digestion was stopped by addition of 0.2 volumes of formic acid and the products were stored at –20 °C.

### High-pressure liquid chromatography (HPLC)

HPLC was performed on a column of CarboPac PA1 (250 × 4 mm; Dionex UK Ltd, https://www.thermofisher.com/uk/en/home/industrial/chromatography/dionex.html) eluted at 1 mL min^–1^ with a linear gradient of 100 % solution A (500 mm NaOH)→100 % solution B (500 mm NaOH in 500 mm sodium acetate) in 30 min followed by isocratic B for 10 min (García-Romera and Fry, 1995) Carbohydrates in the eluate were monitored by use of a pulsed amperometric detector with a gold electrode (Dionex).

### High-voltage paper electrophoresis

Samples of the AIR/Driselase digestion products were loaded as a 20 cm streak (200 µL cm^–1^) on Whatman No. 3 paper. Electrophoresis was conducted at pH 2.0 in a volatile buffer [formic acid/acetic acid/water (1:3.5:35.5 v/v/v)] at 3 kV for 4 h. The apparatus and methods are described by Fry (2020). Papers were dried and viewed under a 254 nm ultraviolet (UV) lamp. A small part of the paper (the fringe of the sample streak plus the whole neighbouring ΔUA–GalA_*n*_ marker mixture) was stained with AgNO_3_ (Fry, 2000).

Unsaturated oligogalacturonides were eluted from specific zones of the unstained part of the paper electrophoretogram in 75 % ethanol, dried and re-dissolved in 50 µL of H_2_O.

### Thin-layer chromatography

Samples eluted from paper electrophoretograms were loaded on TLC plates as 0.8 cm streaks (2.5 µL of each sample). The plate was run in butano1-ol/acetic acid/water (2:1:1) for 7 h, then dried and stained by dipping in thymol solution (0.5 % w/v thymol and 5 % H_2_SO_4_ v/v in ethanol) followed by re-drying and then heating in an oven at 105 °C for 5 min.

### Nuclear magnetic resonance (NMR) spectroscopy

A sample of putative ΔUA–GalA was prepared by complete digestion of 6.6 mg mL^–1^ HG in 3.3 U mL^–1^ PL in 50 mm CAPS (Na^+^, pH 10) and 1 mm CaCl_2_. The resulting ΔUA–GalA was purified by a preparative high-voltage paper electrophoresis, eluted in 75 % ethanol and then dried. The 1-D and 2-D proton and ^13^C-NMR spectra were recorded on a Bruker AVANCE NEO instrument (18.8 T; 800 MHz for protons) using *d*_4_-methanol as solvent. Proton spectra were referenced to the residual CD_2_*H*OD signal at 3.33 ppm and ^13^C spectra were referenced to *C*D_3_OD at 49.0 ppm. Chemical shifts are given in ppm (δ) relative to tetramethylsilane, and scalar coupling constants (*J*) are given in Hz.

### Mass spectrometry

A sample of putative ΔUA obtained by Driselase digestion of de-esterified date fruit cell walls and preparative paper electrophoresis was prepared for electrospray analysis at a concentration of approx. 10 µm in acetonitrile/water (1:1). Analysis was performed on a 12-tesla SolariX 2XR Fourier-transform ion cyclotron resonance (FT-ICR) mass spectrometer (Bruker Daltonics) operating in negative mode. Each spectrum was the sum of 20 scans, with a dataset size of 2 million words. Fragmentation was performed by collision-induced dissociation (CID) with argon as a neutral gas. The collision voltage was 10 V. Data interpretation was achieved with DataAnalysis 5.0 (Bruker Daltonics).

## RESULTS

### *Products formed by action of commercial PL or EPG on commercial HG* in vitro

**Fig. 2.**
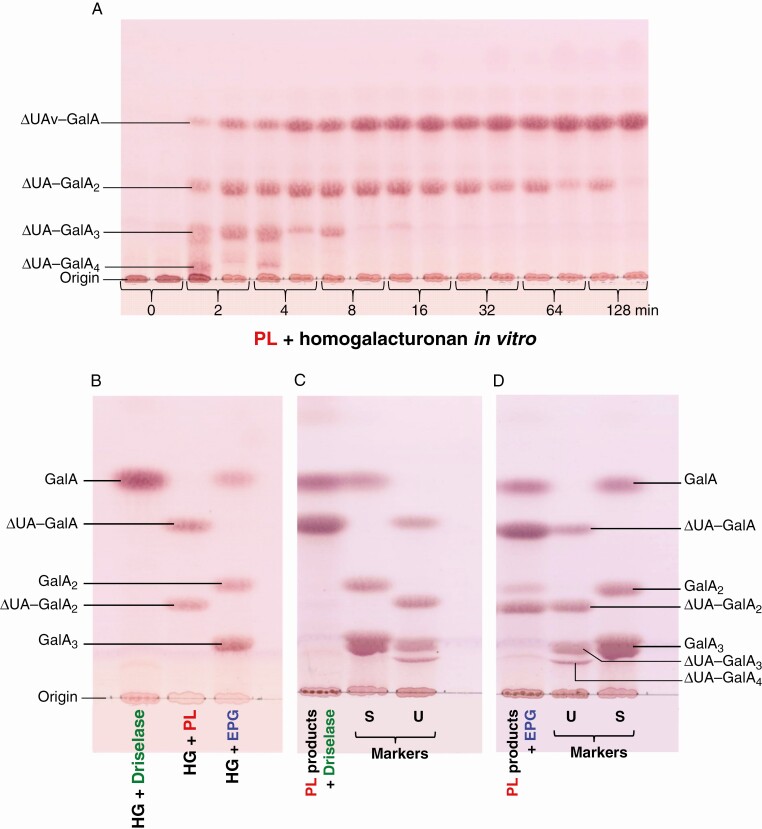
Action of PL on homogalacturonan *in vitro* and further digestion of the products with Driselase or EPG. (A) TLC of products formed from commercial (de-esterified) HG by digestion with commercial PL for 0–128 min. The reaction mixture contained PL at 3.3 U mL^–1^ and the substrate HG at 6.6 mg mL^–1^, in 50 mm CAPS buffer (Na^+^, pH 10.0) with 1 mm CaCl_2_. The reaction was stopped at intervals by addition of 0.2 volumes of formic acid. Each time point is in duplicate, using old and new PL stocks. (B) Evidence that Driselase and commercial EPG lack pectate lyase activity. HG (20 mg mL^–1^) was digested with Driselase (0.05 %, in pyridine/acetic acid/water (1:1:98, v/v/v), pH 4.7) for 3 d, PL (3.3 U mL^–1^, in CAPS/Ca^2+^ as above) for 30 min, or EPG (10 U mL^–1^, in PyAW, pH 4.7) for 16 h, then analysed by TLC. (C and D) Driselase or EPG re-digestion of partial PL digestion products. HG was digested with PL for only 2 min as in (A), then the enzyme was denatured with formic acid and dried *in vacuo*, and the incomplete digestion products were re-digested for 1 week with (C) 0.05 % Driselase at 37 °C or (D) 10 U mL^–1^ EPG at 20 °C, both in PyAW containing 0.05 % chlorobutanol. Marker mixtures were: S, saturated oligogalacturonides; U, unsaturated oligogalacturonides. In all cases: TLC solvent, butan-1-ol/acetic acid/water (2:1:1) with one ascent; stain, thymol.

A time-course for the digestion of commercial HG by commercial PL *in vitro* revealed a range of unsaturated oligogalacturonides even after 2 min at 20 °C, as visualized by TLC (Fig. 2A). The concentration of the smallest product (confirmed below to be a dimer; ΔUA–GalA), indicated by thymol stain intensity, continuously increased with time up to 128 min, by which time the dimer was almost the sole product. The concentration of each of the bigger oligosaccharides transiently peaked and then diminished. A pentasaccharide (ΔUA–GalA_4_), visible at 2 and 4 min, appeared to be the largest product capable of migrating from the origin.

*A priori*, it could be suggested that Driselase or commercial EPG themselves possess PL activity which would generate ΔUA–GalA_*n*_s even from unmodified HG. However, this was shown not to be the case, as Driselase and EPG digestion of commercial HG generated only saturated products. Driselase produced a spot of GalA as the only final product visualized on TLC, and EPG digestion produced GalA, GalA_2_ and GalA_3_ (Fig. 2B).

### Paper electrophoresis separates PL products from EPG products

**Fig. 3.**
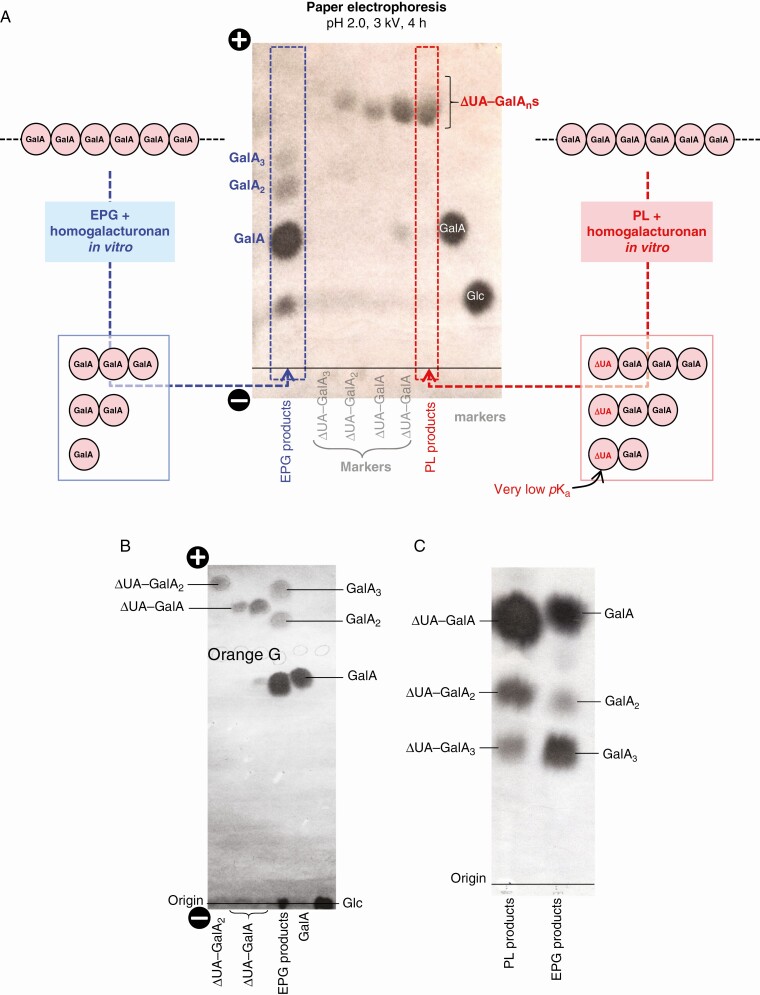
Paper electrophoresis and chromatography for separating PL products from EPG products. (A) Expected and observed products formed from HG by EPG digestion and PL digestion. Left: EPG (10 U mL^–1^) was incubated at 20 °C for 16 h with HG (20 mg mL^–1^) in pyridine/acetic acid/water (1:1:98 v/v/v, containing 0.5 % chlorobutanol), pH 4.7, yielding GalA_3_, GalA_2_ and GalA. Right: PL (3.3 U mL^–1^) was incubated at 20 °C for 10 min with HG (6.6 mg mL^–1^) in 50 mM CAPS buffer (Na^+^, pH 10) containing 1 mM CaCl_2_, yielding ΔUA–GalA_3_, ΔUA–GalA_2_ and ΔUA–GalA. Centre: products were electrophoresed at pH 2.0 (3 kV, 4 h), alongside markers, and stained with AgNO_3_. (Two independent preparations of ΔUA–GalA were run, differing in purity and concentration.) (B) Electrophoresis at pH 6.5 of comparable markers. Markers [left to right: ΔUA–GalA_2_ (PL product); ΔUA–GalA (PL product); GalA_1_, GalA_2_ and GalA_3_ (EPG products); galacturonic acid; glucose] were fractionated by high-voltage paper electrophoresis at pH 6.5 (4 kV, 50 min). Each sample contained an internal marker (Orange G), which was marked in pencil prior to staining. (C) Paper chromatography of comparable markers in ethyl acetate/acetic acid/water (10:5:6) for 30 h.

Paper electrophoresis in pH 2.0 buffer showed a good discrimination between PL and EPG products, providing an efficient method to distinguish the products of these two enzymes. PL products run faster than EPG products owing to the low p*K*_a_ of the ΔUA residue (Fig. 3A). Regardless of the number of GalA residues (ΔUA–GalA_1–3_), PL products ran to a specific region of the electrophoretogram, giving a UV-absorbing spot (characteristic of the ΔUA residue), while EPG products ran slower, with monomeric GalA being the slowest migrating acidic product (Fig. 3A). Electrophoresis at pH 2.0 thus effectively gave a group separation of saturated from unsaturated oligogalacturonides. In contrast, during electrophoresis in pH 6.5 buffer (at which pH all –COOH groups are almost fully ionized; Fry, 2020), GalA_2_ and ΔUA–GalA (which both possess two –COOH groups and are of similar molecular weight) were not well separated (Fig. 3B).The PL and EPG products also overlapped when paper chromatography (Fig. 3C) was used instead of electrophoresis. We therefore recommend electrophoresis at pH 2.0 as the preferred method for isolating PL ‘fingerprints’.

### Driselase trims large PL products to the disaccharide whereas EPG trims them to a mixture of products

The PL products from a brief digestion (2 min) of commercial HG with commercial PL (as in Fig. 2A) followed by either Driselase or EPG digestion showed the smallest product of each. Driselase digestion for up to 1 week at 37 °C produced spots of monomer (GalA) and the unsaturated dimer (ΔUA–GalA), as visualized on TLC (Fig. 2C). On the other hand, EPG digestion for the same period at 20 °C produced a spot of the unsaturated trimer (ΔUA–GalA_2_) in addition to the unsaturated dimer (ΔUA–GalA) plus saturated GalA, GalA_2_ and GalA_3_ (Fig. 2D). Driselase, producing a single unsaturated product, is therefore the preferred agent for isolating a specific PL ‘fingerprint’ (ΔUA–GalA).

### Confirmation of conclusions by HPLC

**Fig. 4.**
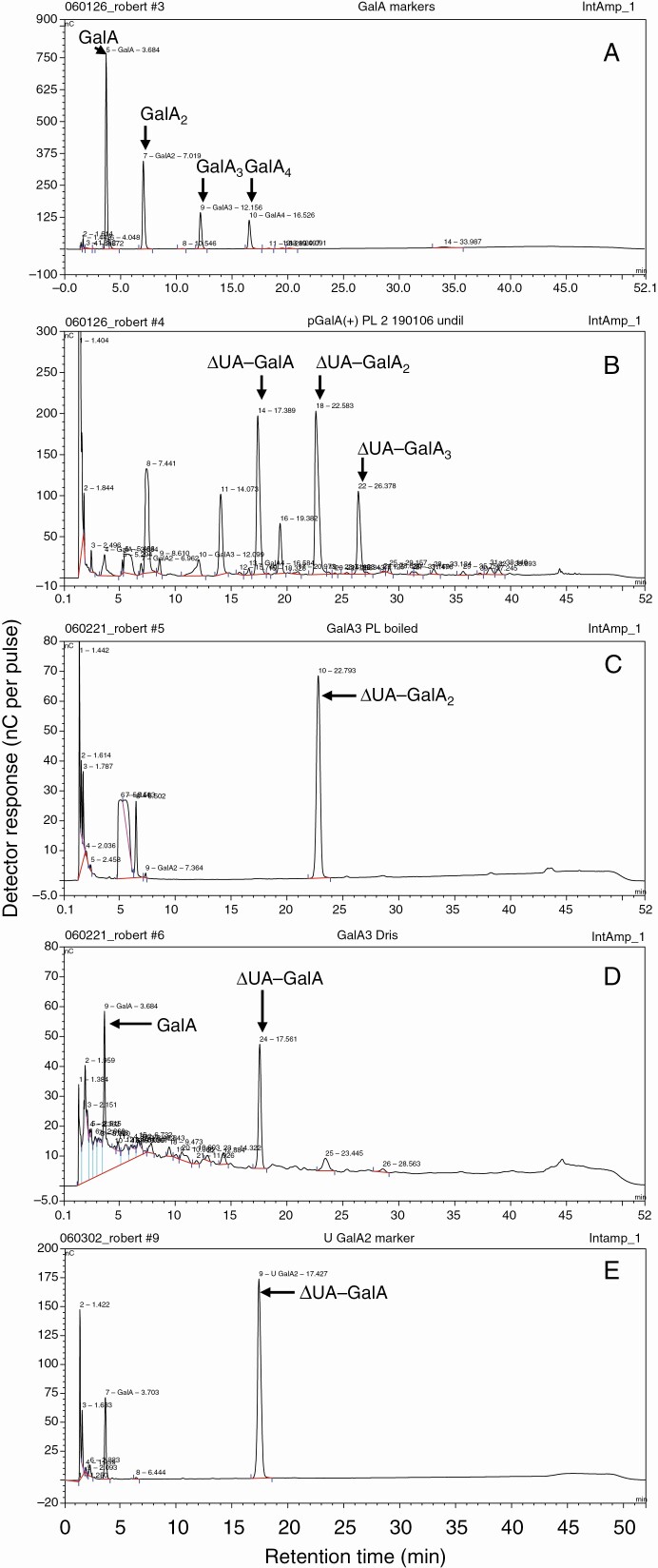
HPLC of products formed by PL action on HG *in vitro* and their further digestion with Driselase. (A) Marker mixture of saturated oligogalacturonides. (B) Unsaturated products of PL action on HG (pH 10.4, 5 min at 37 °C; reaction terminated by 5 min boiling). The unidentified by-products are attributable to the boiling step. (C–E) Monitoring the effect of Driselase on ΔGalA–GalA_2_: (C) ΔGalA–GalA_2_ isolated by partial PL digestion of HG, then treated with denatured Driselase; (D) as in (C) but using active Driselase; (E) marker ΔGalA–GalA. In all cases, HPLC was on a Dionex PA1 HPLC column with a pulsed amperometric detector (gold electrode).

Performing HPLC of the products formed by brief *in-vitro* PL action on HG confirmed the presence of a series of unsaturated oligogalacturonides (Fig. 4B) which did not co-elute with saturated oligogalacturonides (Fig. 4A). The ΔUA–GalA_2_ (Fig. 4C), purified by preparative paper electrophoresis, was digested by Driselase to yield ΔUA–GalA plus free GalA (Fig. 4D).

### NMR evidence for the structure of the proposed ΔUA–GalA

The identity of the proposed ΔUA–GalA, obtained from complete digestion of commercial HG with commercial PL and isolated by preparative high-voltage paper electrophoresis, was tested by NMR spectroscopic analysis.

**Fig. 5.**
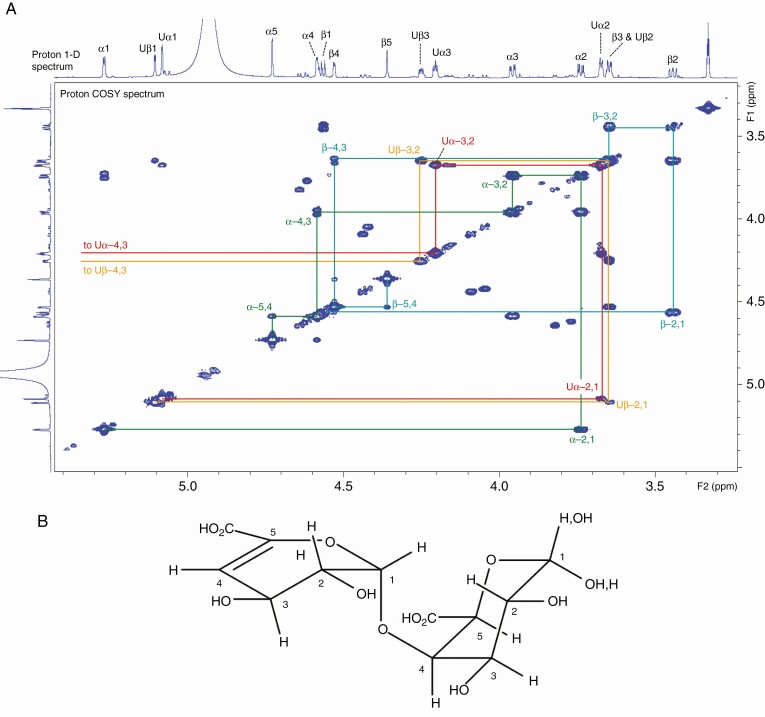
NMR evidence for the structure of the proposed ΔUA–GalA. (A) Proton 1-D and proton COSY NMR spectra (region approximately 3.3–5.4 ppm) of ΔUA–GalA produced by *in-vitro* action of commercial PL on commercial HG. Signals labelled U arise from ΔUA; other labelled signals arise from GalA. The signal at 3.33 ppm arises from CD_2_HOD. (B) Proposed structure of ΔUA–GalA.

The proton spectrum (Fig. 5) showed that the sample of ΔUA–GalA was a mixture of α- and β-anomers (60:40) at GalA. The proton COSY spectrum (Fig. 5) allowed the identification of the separate proton signals. The ^13^C spectrum showed 24 signals as expected. These were assigned from the HSQC 1-bond CH correlation spectrum. Spectral data are given in Table 1. The proton–proton coupling constants confirm the stereochemistry of the GalA residue. The position of the linkage between the two rings is clear from the HMBC spectrum, which showed three-bond correlations between H-1 of ΔUA and C-4 of GalA and between H-4 of GalA and C-1 of ΔUA. All the other signals show correlations between protons and carbons in the same ring. In addition to the expected responses from di-axial protons (close in space), the proton NOESY spectrum also confirmed the presence of the GalA fragment as there are responses between H-3 and H-4, and H-4 and H5, confirming that H-4 is equatorial (H-4 axial would be too far away to give these responses). The response between the ΔUA protons H1 and H2 demonstrates that the linkage there is β-l-. If this were α-l-, these protons would be too far apart to give a response. There are also responses between the H1 of ΔUA and H4 of GalA, supporting the position of linkage on GalA.

**Table 1.** NMR data for ΔUA–GalA

α-anomer (60 %)				β-anomer (40 %)			
	δ _H_	J_HH_ (Hz)	δ _C_		δ _H_	J_HH_ (Hz)	δ _C_
GalA-1α	5.279	3.6	94.4	GalA-1β	4.576	7.8	98.6
GalA-2α	3.750	3.6, 10.2	70.2	GalA-2β	3.454	7.8, 10.0	73.3
GalA-3α	3.969	3.2, 10.2	70.0	GalA-3β	3.658	Obscured	73.7
GalA-4α	4.596	3.2, u	81.4	GalA-4β	4.541	3.1, u	80.2
GalA-5α	4.740	bs	70.7	GalA-5β	4.371	bs	74.3
GalA-6α			172.4	GalA-6β			171.4
ΔUA-1	5.093	1.9	101.5	ΔUA-1	5.116	2.0	101.5
ΔUA-2	3.984	1.9, 5.6	71.8	ΔUA-2	3.658	Obscured	71.9
ΔUA-3	4.216	3.8, 5.6	67.2	ΔUA-3	4.262	3.6, 6.2	67.2
ΔUA-4	6.071	3.8	112.6	ΔUA-4	6.066	3.6	112.9
ΔUA-5			142.7	ΔUA-5			142.5
ΔUA-6			165.3	ΔUA-6			165.4

bs = broad singlet, u = unresolved.

### Detection of PL products in date fruit cell walls

**Fig. 6.**
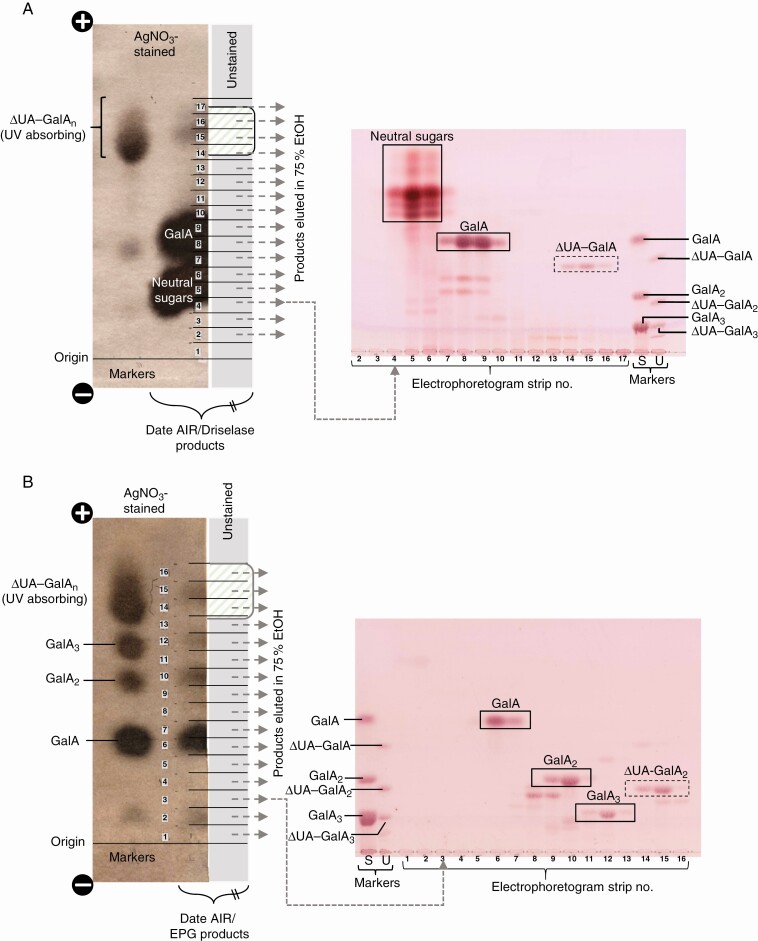
Detecting PL fingerprints in digests of date fruit cell walls by paper electrophoresis and TLC. (A) Driselase digestion. Date AIR (25 mg) was digested in 3 mL of Driselase (0.05 %) in PyAw, containing 0.5 % chlorobutanol at 37 °C. Left: the products were loaded as a 20-cm streak on Whatman No. 3 paper and electrophoresed at pH 2 (3 kV for 4 h). The left-hand fringe of the paper plus the markers were stained with AgNO_3_, visualizing the products. The major portion, only part of which is shown (in grey), was not stained; green/white shading indicates a UV-absorbing band. The whole unstained portion was cut into seventeen 1-cm strips and products were eluted. Right: eluates from strips 2–17 were run by TLC in butan-1-ol/acetic acid/water (2:1:1) alongside marker mixtures, and stained with thymol. Marker mixtures were: S, saturated oligogalacturonides; U, unsaturated oligogalacturonides. (B) EPG digestion. As in (A), but digestion was with EPG (10 U mL^–1^) instead of Driselase.

Using the knowledge gained from the *in-vitro* PL activity experiments, we developed a protocol to detect PL action products *in vivo*. Driselase digestion of de-esterified date fruit cell walls (AIR) would cleave any PL action products, even large products such as ΔUA–GalA_20_, to release the smallest unsaturated product (ΔUA–GalA) plus free GalA. Paper electrophoresis was then used to separate the highly acidic ΔUA–GalA from all other Driselase-generated sugars; TLC then helped to resolve and visualize the ΔUA–GalA, providing the proof for PL action *in viv*. (Fig. 6A).

Paper electrophoresis (pH 2.0) of the products obtained by Driselase digestion of cell walls from ripe dates produced a heavy spot of neutral sugars, a heavy GalA spot and a faster migrating, UV-absorbing spot indicating the presence of highly acidic, unsaturated products (Fig. 6A, left image). The electrophoretogram was cut into transverse strips, eluates of which were analysed by TLC. The neutral fractions (strips 4–6) gave a range of neutral sugars (probably including isoprimeverose, galactose, glucose and rhamnose) (Fig. 6A, right image). Fractions 7–10, which had co-electrophoresed with GalA, were confirmed by TLC to contain predominantly the monosaccharide GalA. TLC of the highly anionic, UV-absorbing fractions (14–16), which had co-electrophoresed with the ΔUA–GalA_*n*_ species, revealed predominantly the dimer, ΔUA–GalA (Fig. 6A), previously shown (Fig. 2C) to be the only unsaturated end-product of Driselase re-digestion of partial PL products.

Driselase digestion of HG (even if pre-digested by EPG) is expected to give only GalA (Fig. 1A, reaction ii), whereas Driselase digestion of PL-pre-treated HG yields in addition one unsaturated dimer, ΔUA–GalA, for every PL event, from the non-reducing terminus (Fig. 1B, reaction ii). Thus the ΔUA–GalA:GalA ratio approximately indicates the number of PL-catalysed cuts per unit chain length of HG. In dates (Fig. 6A), the ΔUA–GalA:GalA ratio was estimated by pixel counting in Photoshop (Vreeburg *et al.*, 2014) to be approx. 1:20, mol mol^–1^, suggesting that roughly one glycosidic bond in 20 of the endogenous HG domains had been cleaved by *in-vivo* PL action in dates. This approximation neglects the GalA generated by Driselase digestion of fruit rhamnogalacturonan-I domains, but remains a reasonable approximation.

Further evidence that the ΔUA residue had been generated by the fruit *in vivo* (rather than artefactually by Driselase) came from a back-up study with commercial EPG, which lacks detectable PL activity (Fig. 2B). When Na_2_CO_3_-de-esterified fruit AIR was exhaustively digested with exogenous EPG, and the products were electrophoresed and fractions analysed by TLC, the major products were, as expected, three (saturated) hydrolysis products: GalA, GalA_2_ and GalA_3_ (Fig. 6B). In addition, a substantial spot of ΔUA–GalA_2_ and a trace of ΔUA–GalA were detected: these electrophoresed with high mobility and ran on TLC in the expected positions. These observations confirm that endogenous PL had been acting *in vivo* on the pectin of live fruit.

### *Mass spectrometric confirmation of the identity of the* in-vivo *PL action product*

**Fig. 7.**
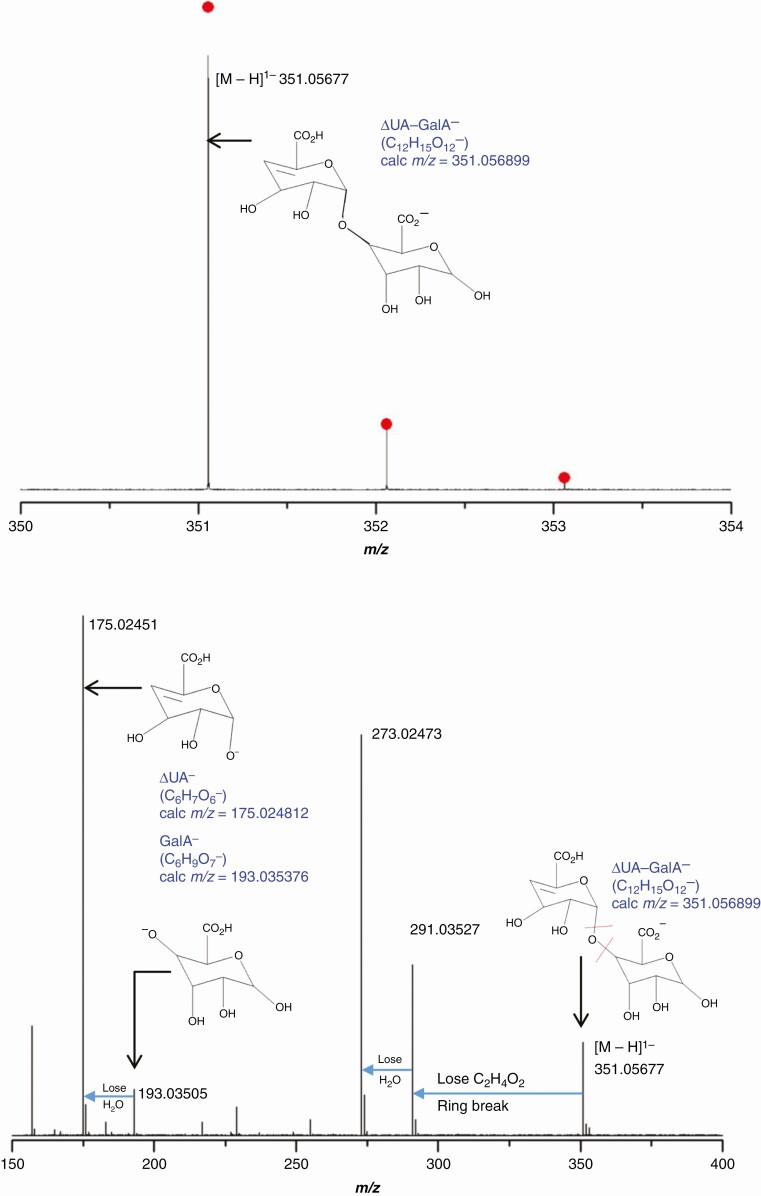
Mass spectrometry of putative ΔUA–GalA obtained by Driselase digestion of de-esterified date fruit cell walls. (A) Negative-mode ESI FT–ICR mass spectrum. The *in-silico* simulated isotope distribution is highlighted (red dots). The mass error is 370 ppb. (B) Negative-mode ESI FT–ICR CID fragmentation mass spectrum of the species identified in (A). Observed *m*/*z* values are labelled in black; proposed identities and their calculated *m*/*z* values are in blue.

Driselase digestion products of de-esterified date AIR were resolved by high-voltage paper electrophoresis as in Fig. 6A. The ΔUA–GalA fraction was then analysed by negative-mode electrospray-ionization FT-ICR mass spectrometry (FT-ICR-MS). The simulated *m/z* of the ∆UA-GalA anion is 351.05690 based on its formula of C_12_H_15_O_12_^−^. Experimentally, molecular-ion negative-mode MS measured the *m/z* at 351.05677, i.e. the value expected with 0.37 ppm error (Fig. 7A).

The CID fragmentation of the ion observed at *m/z* 351.05677 resulted in several fragments that further supported the proposed structure (Fig. 7B).

### PL action products in the Rosaceae

**Fig. 8.**
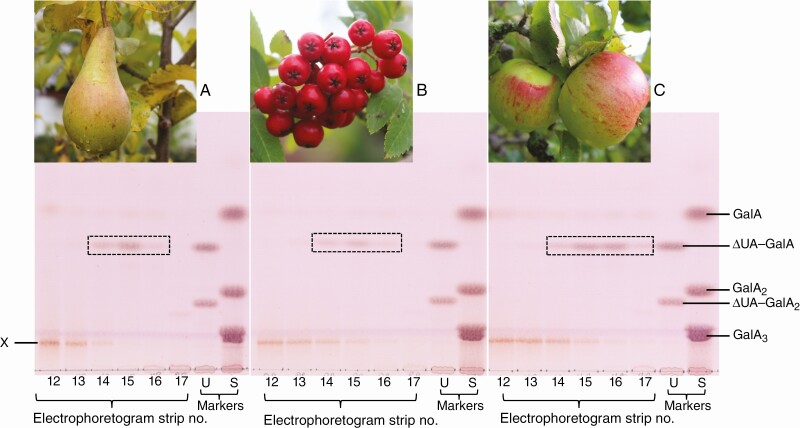
Detecting PL fingerprints in Driselase digests of rosaceous fruit cell walls. AIR from (A) Conference pears, (B) rowan berries and (C) Bramley apples were Driselase digested and analysed as in Fig. 6A. The electrophoretogram fractions expected to contain ΔUA–GalA_*n*_s were subjected to TLC as before. X = unidentified by-product. Marker mixtures are: S, saturated oligogalacturonides; U, unsaturated oligogalacturonides.

Using the methods developed for dates, i.e. *in-vitro* Driselase digestion of de-esterified fruit AIR, we obtained products indicating prior *in-vivo* action of endogenous PL in rowan berries and in two false fruits (pomes: apple and pear). These products were identified by paper electrophoresis and TLC by reference to markers obtained by *in-vitro* digestion of HG with PL or EPG (Fig. 8).

## Discussion

### Wall polysaccharide degradation in fruit: enzyme action contrasted with enzyme activity, gene transcription and protein synthesis

During fruit softening in many species, cell wall composition changes have been reported, especially in pectin domains, mostly presumed to be due to the actions of polysaccharide-modifying enzymes, although additional non-enzymic wall degradation mechanisms can occur (Brummell *et al.*, 1999; Dumville and Fry, 2003; Vreeburg *et al.*, 2014; Airianah *et al.*, 2016). Attention has focused on endo-enzymes, since these cleave polysaccharide molecules in mid-chain, potentially having a greater effect on wall mechanics than exo-enzymes, which only remove single monosaccharide residues. The two endo-enzyme activities that can cleave HG are EPG and PL. While initially reported to be absent (Besford and Hobson, 1972), and later somewhat side-lined, PL is becoming a focus of renewed interest (Marin-Rodriguez *et al.*, 2002; Santiago-Doménech *et al.*, 2008; Wang *et al.*, 2018; Moya-León *et al.*, 2019; Uluisik and Seymour, 2020). PL activity may be difficult to measure in conventional plant extracts *in vitro* as PLs are often deactivated during normal extraction protocols (Payasi *et al.*, 2006); we therefore devised a method for detecting PL action *in vivo*.

A ripening-related increase in extractable PL activity, assayed *in vitro*, was reported in many fruits including tomato (Uluisik *et al.*, 2016), banana (Marín-Rodríguez *et al.*, 2003) and strawberry (Zhou *et al.*, 2016). A suggestion that endogenous PL may exhibit action *in vivo* comes from the observations that, in PL-silenced tomato fruits, less pectin became soluble (Yang *et al.*, 2017) and its molecular weight remained relatively high (Uluisik *et al.*, 2016). However, an unambiguous demonstration of *in-vivo* PL action was lacking. *In-vitro* enzyme activity does not confirm *in vivo* action as there could be restrictions on substrate accessibility, presence of certain inhibitors and/or non-optimum action conditions *in vivo*.

Although gene expression and extractable enzyme activity can suggest that a given enzyme-catalysed reaction could possibly be involved in a physiological process such as ripening, the demonstration of *in-vivo* action of the enzyme remains a gold standard that is difficult to achieve. By quantifying the *in-vivo* action of an enzyme, all transcriptional, post-transcriptional and post-translational modifications are taken into account, together with the regulation of enzyme activity by local cellular environments. In addition to providing a more biologically relevant proof of the *in-vivo* occurrence of polysaccharide modifications, determination of *in-vivo* enzyme action also circumvents problems associated with enzyme denaturation during extraction.

### A strategy for detecting products of PL action

The unique fingerprint of PL action (ΔUA–GalA), described by Fuchs (1965) and Nasuno and Starr (1967), is confirmed in this study. *In-vitro* digestion of HG chains with commercial PL produces oligogalacturonides with an unsaturated non-reducing terminus and a simple galacturonic acid at the reducing terminus (Fig. 1B, reaction i), with the unsaturated dimer (ΔUA–GalA) being the smallest product detected (Fig. 6A). This highly acidic (low p*K*_a_) dimer was separated by electrophoresis at pH 2.0 from all other products (Fig. 3A), to give a sample pure enough for us to prove its identity using TLC (Figs 6A and 8, Supplementary Fig. S1), MS (Fig. 7) and NMR spectroscopy (Fig. 5).

We recommend Driselase rather than EPG for routine analysis of *in-vivo* PL action products because (1) Driselase gave a single unsaturated product (ΔUA–GalA) whereas EPG gave a mixture of ΔUA–GalA_2_ and ΔUA–GalA; (2) EPG gives three saturated oligogalacturonides in addition to the unsaturated ones, whereas the only saturated acidic product of Driselase is the monomer, GalA; and (3) EPG requires the AIR to be pre-saponified, e.g. with Na_2_CO_3_, removing methylester groups, whereas Driselase contains esterases which can remove the methylester groups of HG.

The action of PL in fruit *in vivo* would be unlikely to digest the HG to products as small as ΔUA–GalA or ΔUA–GalA_2_. On the contrary, products of (partial) PL action in fruit would mainly be present in polymeric form (alcohol-insoluble polysaccharides in AIR), making them difficult to isolate and characterize. Therefore, further *in-vitro* hydrolysis of the AIR was performed with Driselase to release a small and well-defined PL action fingerprint, ΔUA–GalA. Driselase was checked to show it has no pectate lyase activity of its own (Fig. 2B) and to be unable to cleave the unsaturated dimer to its monomers (Fig. 2C).

The PL action fingerprint (ΔUA–GalA) was obtained by Driselase digestion of date fruit AIR and documented by electrophoresis and TLC. The mass of the putative ΔUA–GalA isolated from date fruits was confirmed by MS and found to be identical to that of the product obtained *in vitro* by digestion of commercial HG by commercial PL (Fig. 7). The identity of the PL ‘fingerprint’ compound was further confirmed chromatographically and electrophoretically (Figs 6 and 8; Supplementary Fig. S1) and by NMR spectroscopy (Fig. 5).

The action products of PL were also successfully detected by the same strategy in apples, pears and rowan berries (dicots; Rosaceae), confirming that fruit PL action is taxonomically widespread. It is interesting that this contributor to fruit softening was observed both in true fruits (the monocot date and dicot rowan) and in the fleshy parts of false fruits (apple and pear). Another proposed *in-vivo* contributor to fruit softening – apoplastic hydroxyl radicals – was found in true fruits but not in false fruits (Airianah *et al.*, 2016).

Another possible contributor to fruit softening could be rhamnogalacturonan lyase, which non-hydrolytically cleaves rhamnogalacturonan-I in mid-chain. Our finding of pectic polymers possessing ΔUA–GalA termini (the PL fingerprint) concurs with the discovery in cress seed mucilage of the unsaturated disaccharide, ΔUA–rhamnose (lepidimoic acid; Hasegawa *et al.*, 1992), a probable indicator of *in-vivo* rhamnogalacturonan lyase action (Iqbal *et al.*, 2016). It will be interesting to discover whether pectic polymers with ΔUA–rhamnose termini can be detected in fruits, indicating *in-vivo* rhamnogalacturonan lyase action.

### Conclusions

This study deals with wall re-modelling in the living plant cell. Plants express numerous ‘wall-related’ genes, generating mRNAs which, if translated, would encode proteins whose *in silico* predicted enzymic activities suggest that they may be able to re-model the cell wall. In some cases, plant cell walls have been shown to contain the corresponding encoded proteins which, when extracted, exhibit *in-vitro* activity on wall-related polysaccharides. However, in many cases, it remains to be proven that these enzymes exert *in-vivo* action, actually re-modelling the walls of living plant cells. This important question has often been neglected. In the present work, we have developed methods to demonstrate that PL exhibits *in-vivo* action in several fruits. Such action, cleaving the backbone of the pectic HG domain, occurs at the right time and in the right place to play a role in fruit softening. The methods presented open the way to wider documentation of PL action, e.g. in fruits of other species and in non-fruit tissues that also express PL genes, complementing the evidence for *in-vivo* non-enzymic cleavage of polysaccharides by hydroxyl radicals (Airianah *et al.*, 2016).

## SUPPLEMENTARY DATA

Supplementary data are available online at https://academic.oup.com/aob and consist of Figure S1: detecting ΔUA–GalA in Driselase digest of date AIR from three different date samples.

mcab072_suppl_Supplementary_S01

## Notes

Present address: NAK (Nederlandse Algemene Keuringsdienst), Randweg 14, 8304 AS Emmeloord, The Netherlands
