# Supplementary material for: Fruit softening: evidence for pectate lyase action in vivo in date (Phoenix dactylifera) and rosaceous fruit cell walls
Source: Ann Bot. 2021 Jun 10;128(5):511–25. doi: 10.1093/aob/mcab072 (PMC8422893; doi:10.1093/aob/mcab072)
Supplement: mcab072_suppl_Supplementary_S01 [file mcab072_suppl_supplementary_s01.pptx]

## Slide 1
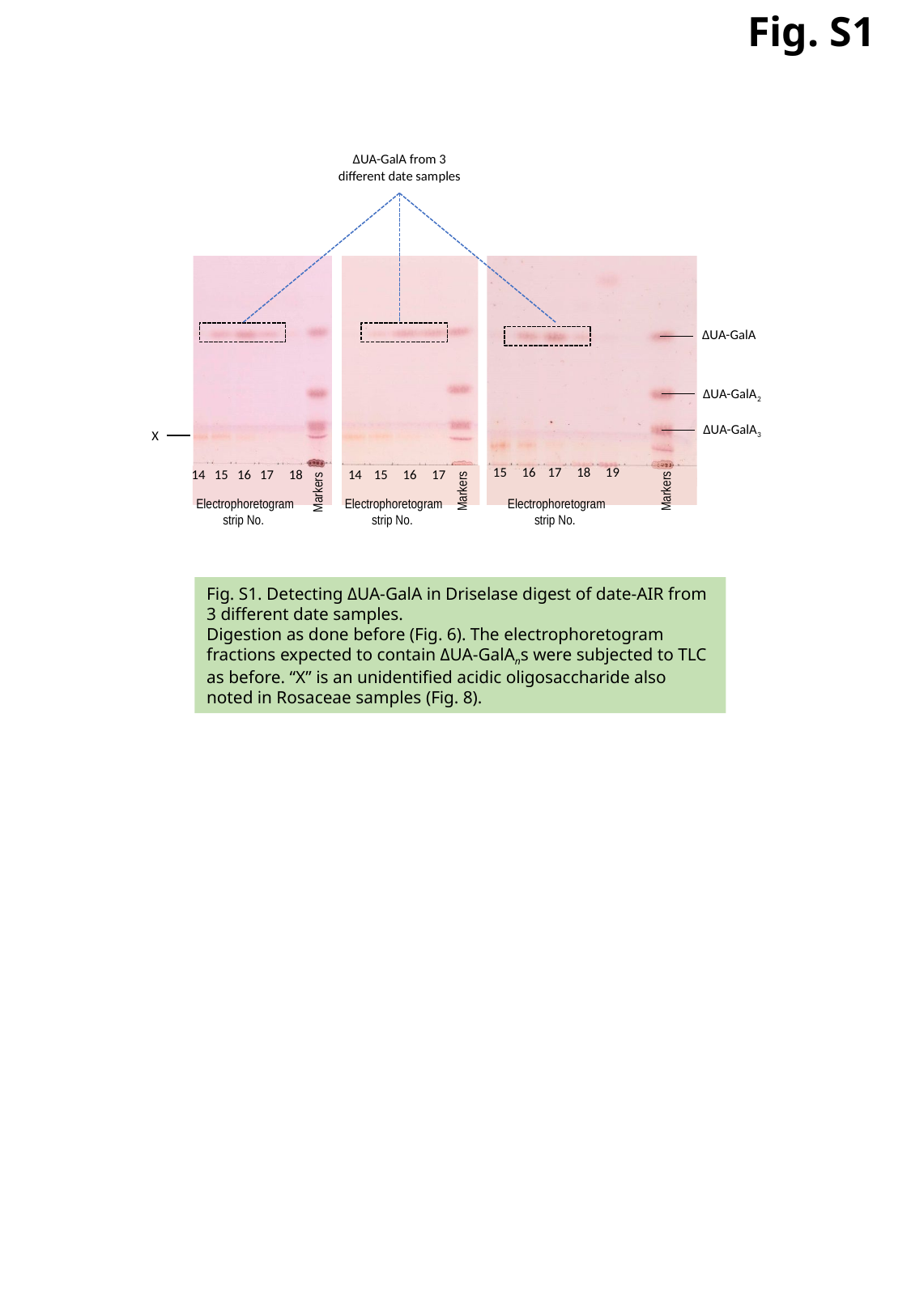

Fig. S1
ΔUA-GalA from 3 different date samples
ΔUA-GalA
ΔUA-GalA2
ΔUA-GalA3
15 16 17 18 19
X
14 15 16 17 18 14 15 16 17
Electrophoretogram strip No.
Electrophoretogram strip No.
Electrophoretogram strip No.
Markers
Markers
Markers
Fig. S1. Detecting ΔUA-GalA in Driselase digest of date-AIR from 3 different date samples.
Digestion as done before (Fig. 6). The electrophoretogram fractions expected to contain ΔUA-GalAns were subjected to TLC as before. “X” is an unidentified acidic oligosaccharide also noted in Rosaceae samples (Fig. 8).
